# High prevalence of ESBL-Producing *E. coli* in private and shared latrines in an informal urban settlement in Dar es Salaam, Tanzania

**DOI:** 10.1186/s13756-017-0292-y

**Published:** 2018-01-06

**Authors:** Stefan Erb, Lauren D’Mello-Guyett, Hamisi M. Malebo, Robert M. Njee, Fatuma Matwewe, Jeroen Ensink, Vladimira Hinic, Andreas Widmer, Reno Frei

**Affiliations:** 1 Division of Infectious Diseases and Hospital Epidemiology, University Hospital Basel, University of Basel, Basel, Switzerland; 2 Environmental Health Group, Faculty of Infectious and Tropical Diseases, London School of Hygiene and Tropical Medicine, London, UK; 3 Maji Safi kwa Afya Bora Ifakara (MSABI), Morogoro, Tanzania; 4 National Institute for Medical Research, Dar es Salaam, Tanzania; 5 Ministry of Health, Community Development, Gender, Elderly and Children, Dar es Salaam, Tanzania; 6 Division of Clinical Microbiology, University Hospital Basel, University of Basel, Basel, Switzerland

**Keywords:** Extended-spectrum beta-lactamase ESBL, Community, Carriage, Prevalence, Latrines, Sub-Saharan Africa

## Abstract

**Background:**

Data about the burden of extended-spectrum beta-lactamase (ESBL)-producing microorganisms in Africa are limited. Our study aimed to estimate the prevalence of human faecal ESBL carriage in the community of an informal urban settlement in Dar es Salaam (Tanzania, East Africa) by using environmental contamination of household latrines with ESBL as a surrogate marker.

**Methods:**

Within the context of a large survey in February 2014 assessing 636 randomly selected household latrines for faecal contamination by the detection of growth of *E. coli* and total faecal coliform bacteria, a randomly selected subset of the samples were screened for ESBL.

**Results:**

Seventy latrines were screened for ESBL. An average of 11.4 persons (SD ±6.5) were sharing one latrine. Only three (4.3%) latrines had hand-washing facilities and 50 showed faeces on the floor. ESBL-producing *Enterobacteriaceae* were confirmed in 17 (24.3%) of the 70 latrine samples: 16 *E. coli* and 1 *Klebsiella pneumoniae.* Five ESBL *E. coli* strains were detected on door handles. The most prevalent ESBL type was CTX-M-1 group (76.5%). Pulsed-field gel electrophoresis typing of a subset of ESBL-producing *E. coli* isolates revealed both diverse singular types and a cluster of 3 identical isolates. There was no significant difference of the latrine and household characteristics between the group with ESBL (*n* = 17) and the group with non-ESBL *E. coli* (*n* = 53) (*p* > 0.05).

**Conclusions:**

Almost a quarter of private and shared latrines in an informal urban settlement in Tanzania are contaminated with ESBL-producing microorganisms, suggesting a high prevalence of human ESBL faecal carriage in the community. Shared latrines may serve as a reservoir for transmission in urban community settings in Tanzania.

## Background

Extended-spectrum beta-lactamase (ESBL)-producing gram-negative bacteria have become an emerging global health threat and have been associated with high mortality [1, 2]. Whereas ESBL infections were initially associated with nosocomial outbreaks, there is now increasing recognition of high rates of faecal carriage and the importance of community-acquired infections due to ESBL-producing *Escherichia coli* in industrialized countries [3, 4]*.*

Data about the prevalence of ESBL-producing microorganisms in Africa are limited. A wide variation from 0.6% up to 77.8% has been reported in hospital-based surveys of clinical isolates [5–11]. There is little published data on the magnitude of the community carriage of ESBL in African countries like Tanzania.

Our study aimed to estimate the prevalence of human faecal ESBL carriage in the community in an urban setting in Tanzania, East Africa, by using environmental contamination of household latrines with ESBL as a surrogate marker.

## Methods

The study was performed in February 2014 in an urban study site in Keko Machungwa, part of the largest unplanned and under-serviced settlement in Temeke district, Dar es Salaam, Tanzania.

**Fig. 1:**
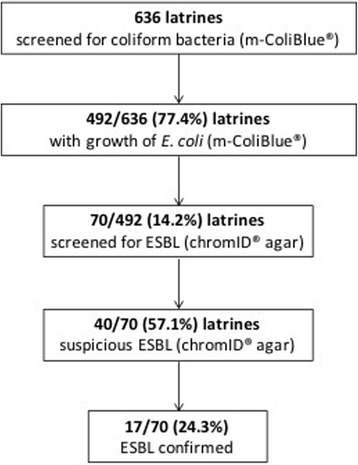
ESBL screening algorithm. ESBL, extended-spectrum beta-lactamase-producing microorganisms

Within the context of a large survey assessing the improvement of sanitation facilities [12], 636 randomly selected household latrines were screened for the presence and concentration of faecal contamination (*E. coli* and total faecal coliform bacteria) [13]. Surface swipe swabs from high frequency contact points (10 cm^2^) like door handle and footrest were taken and analysed using direct membrane filtration technique (Merck Millipore, Billerica, MA, USA) with commercial medium m-ColiBlue® (HACH, Loveland, CO, USA) [14]. In this survey, 492 latrines showed growth of *E. coli* or coliform bacteria either at the footrest, the door handle or both (unpublished data, Fig. 1).

From latrines with *E. coli* growth, samples were selected using a calculator based random number generator and further screened for ESBL-producing *Enterobacteriaceae* using the chromogenic selective culture medium chromID® ESBL (bioMérieux, Marcy-l’Étoile, France) according to the manufacturer’s instructions. ESBL or carbapenemase production was confirmed with standard microbiological techniques following EUCAST guideline [15]. ESBL molecular types (specifically CTX-M-1 and CTX-M-9 groups) were determined by isothermal amplification (eazyplex® SuperBug CRE Assay [Amplex Biosystems, Gießen, Germany] for use on Genie® II platform [Optigene, Horsham, UK]). Antimicrobial susceptibility testing and interpretation was done using Vitek® 2 automated system (bioMérieux) or Etest® (bioMérieux) according to EUCAST clinical breakpoints (version 5.0, 2015; http://www.eucast.org/clinical_breakpoints). Molecular typing was performed by pulsed-field gel electrophoresis (PFGE) as described previously [16].

Data about household and latrine characteristics were collected by visual inspection and questionnaire.

Univariable analysis was performed by the chi-square test or Fisher’s exact test, where appropriate, for categorical variables and two-tailed Student’s t test for continuous variables. Two-tailed *p*-values of <0.05 were considered statistically significant.

The study was approved by the national ethics committee of the National Institute for Medical Research Tanzania (NIMR/HQ/R.8a/Vol.IX/1632).

## Results

From the 492 latrines with growth of *E. coli*, 70 latrines were randomly selected for ESBL screening. All 70 latrines had either a cement, tile or brick floor and met the WHO/UNICEF Joint Monitoring Program for Water Supply and Sanitation definitions for improved latrines [12]. Only 3 (4.3%) latrines had hand-washing facilities and 50 showed faeces on the floor. An average of 11.4 persons (SD ±6.5) were sharing one latrine.

Twenty-four (34.3%) latrines were private (i.e. each of these latrines were used by only one household), the remaining latrines were shared by ≥2 households. The latrines were not accessible for the public but restricted to individuals living in the respective household (Table 1).

**Table 1:** Baseline characteristics of latrines screened for ESBL (*n* = 70)

Characteristics		
Screened latrines (*n*, %)	70	100%
Households sharing one latrine (*n*, %)		
1 houshold (private latrine)	24	34.3%
2 households	26	37.1%
3 households	13	18.6%
4–10 households	4	5.7%
10–23 households	3	4.3%
Persons sharing one latrine (mean, ±SD)	11.4	6.5
Household leader male (*n*, %)	23	32.9%
Household leader’s educational level (*n*, %)		
none	5	7.1%
primary school	43	61.4%
secondary school	22	31.4%
Household leader’s monthly income in US$ (mean, ±SD)	102.7	78.9
Latrines used by children <5 years (*n*, %)	58	82.9%
Latrines with stored bucket for anal cleaning (*n*, %)	26	37.1%
Handwashing facilities <1 m (n, %)	3	4.3%
Soap available in latrine (*n*, %)	6	8.6%
Age of latrines in years (mean, ±SD)	4.6	3.4
Latrine floor material (*n*, %)		
brick	60	85.7%
cement	10	14.3%
Cracked or broken slab (*n*, %)	54	77.1%
Condition of latrine (*n*, %)		
clean	20	28.6%
Dirty - faeces on the floor	50	71.4%
Lid available (*n*, %)	3	4.3%
Latrine separation (*n*,%)		
no door	6	8.6%
curtain	5	7.1%
wood door	59	84.3%
Flies in latrine (*n*, %)	40	57.1%
Animals in the compound (*n*, %)	14	20.0%

*SD* standard deviation

**Fig. 2:**
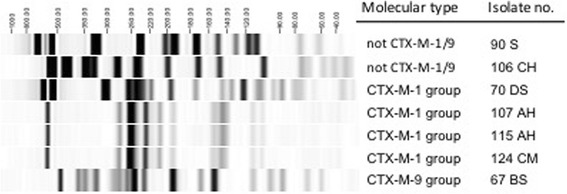
PFGE of seven ESBL-producing *E. coli* isolates. PFGE, pulsed-field gel electrophoresis; ESBL, extended-spectrum beta-lactamase-producing microorganisms. PFGE shows a cluster of 3 isolates (107AH, 115AH, 124CM) with identical PFGE band pattern. Other isolates show different PFGE pattern

Forty samples showed ESBL suspected colonies on chromID® ESBL culture medium. ESBL-producing *Enterobacteriaceae* were finally confirmed in 17 (24.3%) of the 70 latrine samples: 16 *E. coli* and 1 *Klebsiella pneumoniae* (Table 2)*.* Five ESBL *E. coli* strains were detected on door handles. Antimicrobial resistance to ciprofloxacin and trimethoprim/sulfamethoxazole was detected in 94.1% and 82.4% of the ESBL isolates, respectively. No carbapenem resistance or indication for carbapenemase production was detected. The most prevalent ESBL type was CTX-M-1 group (76.5%) (Table 2). Microbiological and household/latrine characteristics of the 17 detected ESBL microorganisms are summarized in Table 3. PFGE typing of a subset of ESBL-producing *E. coli* isolates revealed both diverse singular types and a cluster of 3 identical isolates (Fig. 2). Ten isolates were nontypable by PFGE as observed in other studies [17].

**Table 2:** Microbiological results of the screened latrines (n = 70)

ESBL-producing bacteria total (n, %)	17	24.3%
ESBL *E. coli*	16	
ESBL *K. pneumoniae*	1	
Screening sites positive (n, %)	17	
Footrests	12	70.6%
Door handles ± footrests	5	29.4%
Antibiotics resistant/intermediate^a^ (n, %)		
Ampicillin	17	100%
Amoxicillin/Clavulanic acid	16	94.1%
Piperacillin/Tazobactam	4	23.5%
Cefoxitin	11	64.7%
Ceftazidime	16	94.1%
Ceftriaxone	17	100%
Cefepime	16	94.1%
Ertapenem	0	0%
Meropenem	0	0%
Ciprofloxacin	16	94.1%
Tobramycin	15	88.2%
Amikacin	6	35.3%
Trimethoprim/Sulfamethoxazole	14	82.4%
Nitrofurantoin	1	5.9%
Fosfomycin	0	0%
Colistin	0	0%
ESBL types (n, %)	17	
CTX-M-1 group	13	76.5%
CTX-M-9 group	1	5.9%
Other than CTX-M-1/9 group	3	17.6%

*ESBL* extended-spectrum beta-lactamase-producing microorganisms

^a^Interpretation according to EUCAST breakpoints version 5.0 (2015)

**Table 3:** Microbiological and household characteristics of the 17 detected ESBL microorganisms

No	Sample ID	ESBL organism	ESBL type	Amoxicillin/Clavulanic acid	Piperacillin/Tazobactam	Ceftriaxone	Meropenem	Amikacin	TMP/SMX	Nitrofurantoin	Ciprofloxacin	Fosfomycin	Colistin	Screening site	Age of latrine in years	Persons sharing 1 latrine	Children <5y using 1 latrine	Latrine floor material	Condition of the slab	Condition of the latrine	Lid available	Latrines with stored bucket for anal cleaning	Handwashing facilities	Soap available in latrine	Latrine separation	Flies in latrine	Animals in the compound
1	91BH	*E. coli*	CTX-M-1	R	S	R	S	S	S	S	R	S	S	handle	6	14	3	brick	broken	clean	no	no	no	no	door	yes	yes
2	124CM	*E. coli*	CTX-M-1	R	R	R	S	S	R	S	R	S	S	handle	2	6	1	brick	broken	dirty	no	yes	no	no	door	no	no
3	107AH	*E. coli*	CTX-M-1	R	R	R	S	S	R	S	R	S	S	handle	3	2	0	cement	broken	dirty	no	yes	no	yes	door	yes	yes
4	115AH	*E. coli*	CTX-M-1	R	R	R	S	S	R	S	R	S	S	handle	1	5	1	brick	broken	dirty	no	yes	no	no	door	no	no
5	106CH	*E. coli*	other	R	R	R	S	S	R	S	R	S	S	handle	6	6	1	brick	broken	dirty	no	no	no	no	curtain	no	yes
6	104DS	*E. coli*	other	R	S	R	S	S	R	S	R	S	S	floor	0.5	15	1	brick	no cracks	dirty	no	no	no	no	door	yes	no
7	102DS	*E. coli*	CTX-M-1	R	S	R	S	R	R	S	R	S	S	floor	4	15	1	brick	broken	dirty	no	no	no	no	door	yes	no
8	90S	*E. coli*	other	R	S	R	S	S	R	S	R	S	S	floor	4	15	1	brick	broken	dirty	no	no	no	no	door	no	no
9	86BS	*E. coli*	CTX-M-1	R	S	R	S	S	R	S	R	S	S	floor	0.5	5	1	cement	broken	dirty	yes	yes	yes	yes	door	no	no
10	84BS	*E. coli*	CTX-M-1	R	S	R	S	R	R	S	R	S	S	floor	0.5	23	1	brick	broken	clean	no	no	no	no	door	yes	no
11	83BS	*E. coli*	CTX-M-1	R	S	R	S	R	R	S	R	S	S	floor	0.5	12	2	brick	no cracks	clean	no	no	no	no	door	yes	no
12	97DS	*E. coli*	CTX-M-1	R	S	R	S	R	R	S	R	S	S	floor	2	9	1	cement	broken	dirty	no	yes	no	no	door	no	yes
13	93AS	*E. coli*	CTX-M-1	R	S	R	S	R	R	S	R	S	S	floor	0.5	10	1	brick	broken	dirty	no	yes	no	no	door	no	yes
14	70DS	*E. coli*	CTX-M-1	R	S	R	S	S	S	S	R	S	S	floor	1	15	4	brick	broken	dirty	no	no	no	no	door	yes	no
15	67BS	*E. coli*	CTX-M-9	R	S	R	S	S	R	S	R	S	S	floor	2	4	1	brick	broken	clean	no	no	no	no	no	yes	no
16	94AS	*E. coli*	CTX-M-1	R	S	R	S	R	R	S	R	S	S	floor	9	7	0	cement	broken	dirty	yes	no	no	no	curtain	yes	no
17	64BA	*K. pneumoniae*	CTX-M-1	S	S	R	S	S	S	R	S	S	S	floor	6	10	1	brick	no cracks	clean	no	no	no	no	door	yes	yes

Susceptibility testing according to EUCAST: *R* resistant or intermediate, *S* susceptible, *TMP/SMX* Trimethoprim/Sulfamethoxazole

There was no significant difference of the latrine and household characteristics between the group with ESBL (*n* = 17) and the group with non-ESBL *E. coli* (*n* = 53) (*p* > 0.05).

## Discussion

In this study, we found that a quarter of the *E. coli* from contaminated latrines in an urban informal settlement in Dar es Salaam express ESBL, suggesting a high prevalence of human ESBL faecal carriage in the community, and that shared latrines may serve as a reservoir for transmission in urban areas of Tanzania.

In a recent meta-analysis, the median proportion of ESBL-producing *Enterobacteriaceae* in patients of Tanzanian healthcare facilities was 39% (range 14.2–75.9%) [11]. Only little is known about the ESBL burden in a community setting in Tanzania. Tellevik et al. reported an ESBL faecal carriage rate in healthy community children in Dar es Salaam of 11.6% [18]. An elderly survey from 2004 found ESBL in 16% of Enterobacteriaceae causing community-acquired urinary tract infections [19]. Studies from other African countries like Senegal, Niger and Madagascar have reported ESBL carriage rates in the community ranging as high as 10% to 31% [3]. In cases where they have been identified, CTX-M enzymes were predominantly of CTX-M-1 group as in our isolates.

Community-onset infections with ESBL-producing pathogens are now increasingly reported. Empirical and targeted antibiotic treatment of such infections is challenging particularly in resource-limited countries. ESBL producing gram-negative bacteria are by definition resistant to extended-spectrum cephalosporins and frequently carry additional resistance genes conferring reduced susceptibility to many other antibiotics like e.g. fluoroquinolones. In many cases the carbapenems remain the only choice for treatment of infections caused by these resistant bacteria. However, the access to antimicrobial agents active against ESBL is limited in many regions of East Africa, thus common infections with multidrug-resistant pathogens cannot be treated adequately.

Intestinal colonization with ESBL-producing microorganisms may last several weeks, months or even years and is a potential source for human-to-human transmission. Precarious hygienic conditions of latrines, which are commonly shared amongst different households and household members, and the presence of ESBL-producing microorganisms on door handles may enable further spread. The cluster of 3 identical *E. coli* strains from 3 different latrines detected by PFGE typing in our study points to the potential of ESBL transmission in the community.

Our study has limitations: 1) Due to ethical and logistical reasons rectal swabs were not feasible. However, environmental samples of the latrines may give a valuable estimate of the frequency and distribution of ESBL-producing microorganisms. In addition, there is a lower risk of selection bias in the community and the results provide information about potential transmission pathways of multidrug-resistant pathogens in the community, similar to a recent study from airport toilet door handles [20]. 2) Our study was restricted to one informal urban settlement in Dar es Salaam and might differ from other urban and rural settings in Tanzania and East Africa. 3) Due to financial constraints only 70 latrines could be screened for ESBL.

## Conclusions

Our study provides evidence of a high prevalence of human ESBL faecal carriage in the community of a resource-limited country such as Tanzania. Further larger surveillance studies are needed in Africa to better describe the epidemiology of ESBL, to raise awareness of the need of strategies to prevent further dissemination, and to improve the access and responsible use of appropriate antimicrobial agents in the empirical treatment of infections caused by otherwise deadly multidrug-resistant microorganisms.

## References

[1] WHO. Antimicrobial resistance: Global report on surveillance. Geneva: World Health Organization; 2014.

[2] Schwaber MJ, Carmeli Y (2007). Mortality and delay in effective therapy associated with extended-spectrum b-lactamase production in Enterobacteriaceae bacteraemia: a systematic review and meta-analysis. J Antimicrob Chemother.

[3] Woerther PL, Burdet C, Chachaty E, Andremont A (2013). Trends in human fecal carriage of extended-spectrum beta-lactamases in the community: toward the globalization of CTX-M. Clin Microbiol Rev.

[4] Ny S, Lofmark S, Borjesson S (2017). Community carriage of ESBL-producing 253 Escherichia coli is associated with strains of low pathogenicity: A Swedish 254 Nationwide Study. J Antimicrob Chemother.

[5] Frank T, Arlet G, Gautier V, Talarmin A, Bercion R (2006). Extended-spectrum beta-lactamase-producing Enterobacteriaceae, Central African Republic. Emerg Infect Dis.

[6] Moyo SJ, Aboud S, Kasubi M, Lyamuya EF, Maselle SY (2010). Antimicrobial resistance among producers and non-producers of extended spectrum beta-lactamases in urinary isolates at a tertiary hospital in Tanzania. BMC Res Notes.

[7] Mshana SE, Matee M, Rweyemamu M (2013). Antimicrobial resistance in human and animal pathogens in Zambia, Democratic Republic of Congo, Mozambique and Tanzania: an urgent need of a sustainable surveillance system. Ann Clin Microbiol Antimicrob.

[8] Tansarli GS, Poulikakos P, Kapaskelis A, Falagas ME (2014). Proportion of extended-spectrum beta-lactamase (ESBL)-producing isolates among Enterobacteriaceae in Africa: evaluation of the evidence - systematic review. J Antimicrob Chemother.

[9] Sangare SA, Maiga AI, Guindo I (2015). Prevalence of extended-spectrum beta-lactamase-producing Enterobacteriaceae isolated from blood cultures in Africa. Med Mal Infect.

[10] Storberg V. Esbl-producing Enterobacteriaceae in Africa - a non-systematic literature review of research published 2008–2012. Infect Ecol Epidemiol. 2014;4. 10.3402/iee.v4.20342. eCollection 2014. 10.3402/iee.v4.20342 PMC3955770 24765249

[11] Sonda T, Kumburu H, van Zwetselaar M (2016). Meta-analysis of proportion estimates of extended-spectrum-beta-lactamase-producing Enterobacteriaceae in East Africa hospitals. Antimicrob Resist Infect Control.

[12] Bain RE, Gundry SW, Wright JA, Yang H, Pedley S, Bartram JK (2012). Accounting for water quality in monitoring access to safe drinking-water as part of the millennium development goals: lessons from five countries. Bull World Health Organ.

[13] Exley JL, Liseka B, Cumming O, Ensink JH (2015). The sanitation ladder, what constitutes an improved form of sanitation?. Environ Sci Technol.

[14] Rusin P, Orosz-Coughlin P, Gerba C. Reduction of faecal coliform, coliform and heterotrophic plate count bacteria in the household kitchen and bathroom by disinfection with hypochlorite cleaners. J Appl Microbiol 1998; 85: 819-828. 10.1046/j.1365-2672.1998.00598.x 9830117

[15] EUCAST. Eucast guideline for the detection of resistance mechanisms and specific resistances of clinical and/or epidemiological importance. Version 1.0. European Committee on Antimicrobial Susceptibility Testing .2013. http://www.eucast.org/resistance_mechanisms/.

[16] Pfaller M, Hollis R, Sader H (1992). Chromosomal restriction fragment analysis by pulsed-field gel electrophoresis.

[17] Oethinger M, Conrad S, Kaifel K (1996). Molecular epidemiology of fluoroquinolone-resistant Escherichia Coli bloodstream isolates from patients admitted to European cancer centers. Antimicrob Agents Chemother.

[18] Tellevik MG, Blomberg B, Kommedal O, Maselle SY, Langeland N, Moyo SJ (2016). High prevalence of faecal carriage of ESBL-producing Enterobacteriaceae among children in Dar es Salaam, Tanzania. PLoS One.

[19] Manyahi J, Moyo SJ, Tellevik MG (2017). Detection of CTX-M-15 beta-lactamases in Enterobacteriaceae causing hospital- and community-acquired urinary tract infections as early as 2004, in Dar es Salaam, Tanzania. BMC Infect Dis.

[20] Schaumburg F, Kock R, Leendertz FH, Becker K (2016). Airport door handles and the global spread of antimicrobial-resistant bacteria: a cross sectional study. Clin Microbiol Infect.

